# Human *Borrelia miyamotoi* Infection in North America

**DOI:** 10.3390/pathogens12040553

**Published:** 2023-04-03

**Authors:** Jed Burde, Evan M. Bloch, Jill R. Kelly, Peter J. Krause

**Affiliations:** 1 Department of Epidemiology of Microbial Diseases, Yale School of Public Health, New Haven, CT 06520, USA; 2 Division of Transfusion Medicine, Department of Pathology, Johns Hopkins University, Baltimore, MD 21217, USA; 3 Department of Environmental Health Sciences, Yale School of Public Health, New Haven, CT 06520, USA; 4 Department of Medicine, Yale School of Medicine, New Haven, CT 06510, USA

**Keywords:** *Borrelia miyamotoi*, relapsing fever, epidemiology, emerging infection, ticks, human

## Abstract

*Borrelia miyamotoi* is an emerging pathogen that causes a febrile illness and is transmitted by the same hard-bodied (ixodid) ticks that transmit several other pathogens, including *Borrelia* species that cause Lyme disease. *B. miyamotoi* was discovered in 1994 in *Ixodes persulcatus* ticks in Japan. It was first reported in humans in 2011 in Russia. It has subsequently been reported in North America, Europe, and Asia. *B. miyamotoi* infection is widespread in *Ixodes* ticks in the northeastern, northern Midwestern, and far western United States and in Canada. In endemic areas, human *B. miyamotoi* seroprevalence averages from 1 to 3% of the population, compared with 15 to 20% for *B. burgdorferi*. The most common clinical manifestations of *B. miyamotoi* infection are fever, fatigue, headache, chills, myalgia, arthralgia, and nausea. Complications include relapsing fever and rarely, meningoencephalitis. Because clinical manifestations are nonspecific, diagnosis requires laboratory confirmation by PCR or blood smear examination. Antibiotics are effective in clearing infection and are the same as those used for Lyme disease, including doxycycline, tetracycline, erythromycin, penicillin, and ceftriaxone. Preventive measures include avoiding areas where *B. miyamotoi*-infected ticks are found, landscape management, and personal protective strategies such as protective clothing, use of acaricides, and tick checks with rapid removal of embedded ticks.

## 1. Epidemiology

### 1.1. Introduction

Scientists discovered a new *Borrelia* species in 1994 while screening *Ixodes persulcatus* ticks and *Apodemus argenteus* field mice for *Borrelia* infection in Hokkaido, Japan [1]. The microorganism was named *Borrelia miyamotoi* in honor of Kenji Miyamoto, an entomologist who was the first to isolate spirochetes from hard-bodied ticks in Japan. Phylogenetic analysis confirmed that this microorganism belongs to the relapsing fever *Borrelia* group. This is surprising because relapsing fever *Borrelia* are characteristically transmitted by soft-bodied (argasid) ticks, while Lyme disease *Borrelia* are transmitted by hard-bodied (ixodid) ticks [2,3]. The discovery of *B. miyamotoi*-infected *I. scapularis* ticks in Connecticut, USA confirmed that these bacteria were transmitted by *Ixodes* ticks and that it had a broad geographic distribution [4]. *B. miyamotoi* was thought to be an endosymbiont until 2011 when human *B. miyamotoi* infection was described by Platonov and colleagues in 46 residents of Yekaterinburg City, Russia [5]. All patients experienced a viral-like illness and about a tenth had relapsing fever. Relapsing fever may have been more common had the patients not been treated with antibiotic relatively early in the course of their disease. Numerous cases and serological evidence of past *B. miyamotoi* infection in humans subsequently were described in North America, Europe, and Asia [6,7,8,9,10,11]. Genotypic and phenotypic features of *B. miyamotoi* that are characteristic of relapsing fever *Borrelia* include transstadial and transovarial tick transmission, generation of a higher *Borrelia* burden in the blood than in the skin, relapsing fever, and a glycerophosphodiester phosphodiesterase (GlpQ) protein that is found in relapsing fever *Borrelia* but not in Lyme disease *Borrelia* [12,13,14,15,16]. *B. miyamotoi*-infected female ticks can pass the infection to their eggs, so that some larval ticks are infected and can then transmit the infection to a vertebrate host (transovarial transmission) [17,18]. Other larvae become infected after taking a blood meal on an infected mouse reservoir host, molt to the nymphal stage, and then transmit infection to another mouse or human (transstadial transmission). Transovarially infected larvae may be the primary source of *B. miyamotoi* infection in vertebrate hosts [18]. The presence of high blood concentrations of *B. miyamotoi* on blood smear and the detection of antibodies in a GlpQ-based antibody assay help to confirm *B. miyamotoi* infection and distinguish it from Lyme disease [7,8,14]. In this review, we focus on the epidemiology and clinical manifestations of *B. miyamotoi* in North America. We discuss the frequency and location of infection in ticks and people, clinical presentation and complications, diagnosis, treatment, and prevention.

### 1.2. The Organism

*B. miyamotoi* is a relapsing fever spirochete in the genus *Borrelia*. *Borrelia* is divided into two main clades, the *Borrelia burgdorferi* sensu lato group and the relapsing fever group. The *B. burgdorferi* sensu lato group contains 20 species, including the causative agents of Lyme borreliosis, and are solely transmitted by hard-bodied ticks. The relapsing fever group consists of 25 species that includes *Borrelia miyamotoi* [10,11]. Most of the relapsing fever species are transmitted by soft-bodied ticks but some species are transmitted by hard-bodied ticks (*B. miyamotoi*, *Borrelia lonestari*, *Borrelia theileri*) or by lice (*Borrelia recurrentis*). *B. miyamotoi* shares phenotypic characteristics of the relapsing fever group such as relapsing fever, a high level of spirochetemia in blood, and transovarial transmission; however, it also has some characteristics of the *B. burgdorferi* sensu lato group, most notably transmission by hard-bodied ticks. [7,8,10,13,19].

Phylogenetic analyses of the *B. miyamotoi* genome have provided important insights into the differences between *B. miyamotoi*, the Lyme disease *Borrelia*, and other relapsing fever *Borrelia* [12,19,20,21,22,23,24,25,26,27,28]. A complete genomic sequencing revealed that the genome consists of 1362 genes on one linear chromosome and 12 linear and 2 circular plasmids [26,27]. There are genetic similarities between *B. miyamotoi* and other relapsing fever *Borrelia* spp., as reflected by broadly cross-reacting antibodies that may complicate diagnostic identification of *B. miyamotoi* from other relapsing fever species [29]. At the same time, there is strong evidence of genetic differences between *B. miyamotoi* and other relapsing fever *Borrelia* and between different *B. miyamotoi* isolates. Genetic analysis of *B. miyamotoi* tick isolates suggest that these microorganisms belong to a species complex (*B. miyamotoi* sensu lato) with Asian, European, and North American genotypes. Differences between isolates may not be due to geographic location, however, but rather to vector competence or host range [8,11,19,27,28]. Further investigation is needed to confirm this hypothesis. Genes that encode for variable membrane proteins (VMPs) have been found in various plasmids of *B. miyamotoi* [24,27]. Similar gene families in other relapsing fever species trigger change in outer membrane proteins during the course of infection as a host immune evasion strategy. These changes render antibodies that are directed against previous outer membrane proteins of the microorganism ineffective against the new gene-altered outer membrane. Recurrent clinical relapses are characteristic of relapsing fever *Borrelia* infections. Another relapsing fever *Borrelia* gene of interest is the glycerophosphodiester phosphodiesterase (GlpQ) biosynthetic gene that is absent in *B. burgdorferi* species [14]. The presence of antibodies against *B. miyamotoi* GlpQ antigen thus helps to distinguish illness due to *B. miyamotoi* infection from that due to *B. burgdorferi* [7,14,16,30].

### 1.3. Ecology

**Figure 1:**
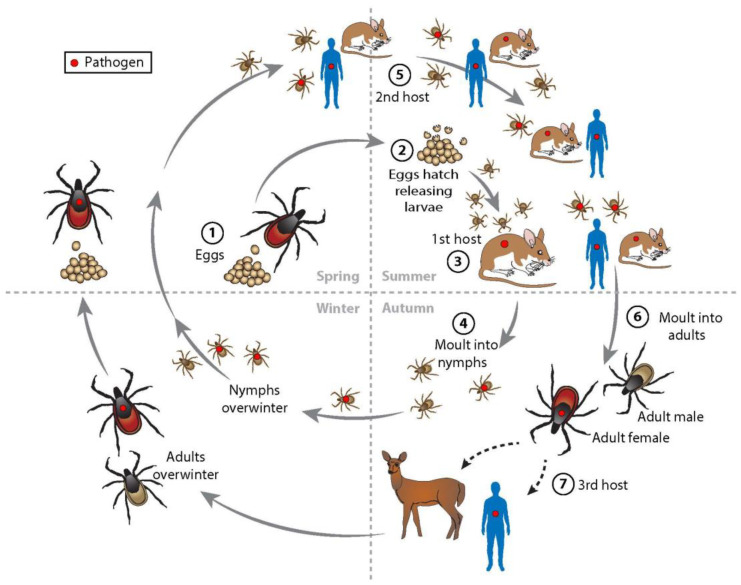
*Ixodes scapularis* life cycle. Female *I. scapularis* lay eggs in the spring that hatch in early summer and produce larvae (1 and 2). Larval *I. scapularis* ticks become infected with *B. miyamotoi* when they take a blood meal from infected white-footed mice (*Peromyscus leucopus*) or other small rodent hosts in late summer (3). Larvae molt into nymphs and overwinter (4). The following late spring, summer, and early autumn, infected nymphs transmit *B. miyamotoi* to uninfected mice or humans when they take a blood meal (5). During the following autumn, nymphs molt into adults (6). Adults feed on white-tailed deer (*Odocoileus virginianus*) but rarely on humans (7). Adults overwinter and the females lay eggs in the early spring to complete the tick life cycle. White-tailed deer amplify the tick population by providing a breeding site for male and female ticks and a blood meal that allows female ticks sufficient protein to lay eggs in the spring (adapted from New England Journal of Medicine, Vannier E., Krause P.J. Human babesiosis. 2012; 366: 2399. Copyright 2019 Massachusetts Medical Society. Reprinted with permission) [32].

*Borrelia miyamotoi* is transmitted to humans by *Ixodes* ticks after they acquire the spirochete from animal reservoirs (Figure 1). Four tick species account for most transmission: *Ixodes scapularis* and *Ixodes pacificus* in North America, *Ixodes ricinus* in Europe, and *Ixodes persulcatus* in Eurasia. *Ixodes ovatus* and *Ixodes pavlovskyi* transmit *B. miyamotoi* in northern Asia. An analysis of 101 studies of *B. miyamotoi* infection in 165,637 questing ticks revealed average infection values of 2.8% (95% CI 2.4–3.1%) in *I. persulcatus*, 1.1% (95% CI 1.0–1.2%) in *I. scapularis*, 1·0% (95% CI 1.0–1.1%) in *I. ricinus*, and 0.7% (95% CI 0.6–0.8%) in *I. pacificus* [6]. There is marked variability in tick infection frequencies, which may be as high as 8.9% for *I. persulcatus*, 5.5% for *I. scapularis*, and 3.8% for *I. ricinus*. Tick infection values of *B. miyamotoi* are approximately 5- to 20-fold less than for the agents of Lyme disease, anaplasmosis, and babesiosis. Coinfections occur in reservoir hosts, ticks, and humans with up to four pathogens infecting a single *I. scapularis* tick [31]. 

There are three active stages in the *I. scapularis* tick life cycle (larva, nymph, and adult). Each takes a blood meal from a vertebrate host in order to mature to the next stage (Figure 1). Once ingested, *B. miyamotoi* travels to the salivary glands and then may be transmitted to a new, uninfected reservoir host. That host then serves as a source of infection for uninfected ticks [33]. Sequential transmission from one life stage to the next (e.g., from larvae to nymph) is known as transstadial passage. *B. miyamotoi* also may invade the ovaries of a gravid *I. scapularis* female and pass transovarially to larvae. When larvae transmit the infection to vertebrates, all three tick life cycle stages transmit infection [17,18]. Although larvae, nymphs, and adults can feed on humans, nymphs are the primary vector for most *Ixodes*-transmitted pathogens. Interestingly, transovarially-infected larvae may be the most common tick stage transmitting *B. miyamotoi* infection [18,33]. Adult ticks preferentially feed on white-tailed deer (*Odocoileus virginianus*). An increase in the deer population during the past few decades is thought to be a major factor in the spread and increased number of *I. scapularis* ticks and the resulting increase in human *I. scapularis*-transmitted infections [33]. Although the reservoir hosts of *B. miyamotoi* are uncertain or unknown throughout much of its distribution, the white-footed mouse (*Peromyscus leucopus*) appears to be the most common reservoir host in the United States [4,33,34]. Other potential reservoir species include birds, different species of field mice, and voles [35,36,37]. 

### 1.4. Location and Prevalence

#### 1.4.1. USA

**Figure 2:**
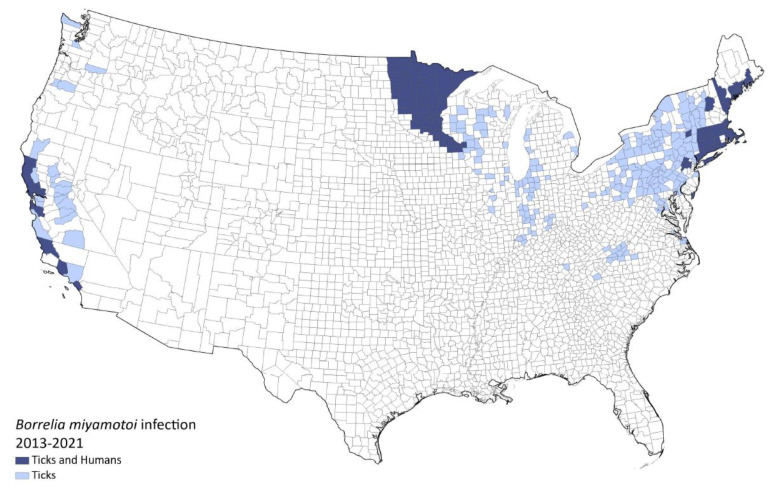
*Borrelia miyamotoi* infection in *Ixodes* ticks and humans by county in the USA 2013–2021.

*B. miyamotoi* infection is widespread in *Ixodes scapularis* ticks in the northeastern, northern Midwestern, and western United States [4,34,38,39,40,41,42,43,44,45,46,47,48,49,50,51,52,53,54,55,56,57,58,59,60,61,62,63,64,65,66,67,68,69] (Figure 2). We analyzed the combined results of multiple studies of field collected nymphal *I. scapularis* ticks that were tested for both *B. miyamotoi* and *B. burgdorferi* between 1998 and 2019. An average of 1.5% ticks were infected with *B. miyamotoi*, and 20.4% were infected with *B. burgdorferi* in the Northeast (Table 1) [4,34,38,39,40,41,42,43,44,45,46,47]. Studies carried out in *I. scapularis* ticks in the Midwest and *I. pacificus* ticks in the far West from 2000 to 2016 showed an average of 2.1% and 1.2% *B. miyamotoi*-infected nymphal ticks, and 17.7% and 3.9% *B. burgdorferi*-infected nymphal ticks, respectively (Table 1) [34,38,39,48,49,50,51,52,53,54,55,56,57,58,59,60]. In contrast, the average human *B. miyamotoi* and *B. burgdorferi* seroprevalence of residents of the Northeast enrolled in four serosurveillance studies was 3.0% (0.6–5.3%) for *B. miyamotoi* and 10.8% (6.8–15.6%) for *B. burgdorferi* [16,59,70,71]. 

#### 1.4.2. Canada

*B. miyamotoi*-infected *Ixodes* ticks have been detected in all Canadian provinces except Newfoundland and Labrador [72,73,74,75,76,77,78,79,80,81,82,83]. Among studies where both *B. miyamotoi* and *B. burgdorferi* infection were found at the same study sites in nymphal ticks, an average of 0.6% of ticks were infected with *B. miyamotoi* and 18.9% with *B. burgdorferi* [71,77]. No human *B. miyamotoi* cases have yet been reported in Canada, but a serosurvey of 10,000 blood donors in Manitoba revealed that 3% were seropositive for *B. miyamotoi* [81]. An increase in the tick vector population and its geographic range in Canada has been accompanied by a marked increase in Lyme disease cases. Warmer temperatures due to climate change have been implicated as a major cause of the northern expansion of *I. scapularis* ticks in North America [79,80,81,82]. A similar increase can be anticipated for human *B. miyamotoi* infection. 

**Table 1:** *B. miyamotoi* and *B. burgdorferi* infection in nymphal *Ixodes* ticks and humans in North America.

Survey	Location	Dates	*B. miyamotoi*-Infected Average % (Range)	*B. burgdorferi*-Infected Average % (Range)
Human infection [16,59,70,71] (seroprevalence)	USA Northeast	1991–2018	3.0 (0.6–5.2)	10.8 (6.8–15.6)
*I. scapularis* infection [4,34,38,39,40,41,42,43,44,45,46,47]	USA Northeast	1998–2019	1.5 (0–10.5)	20.4 (2.6–49.7)
*I. scapularis* infection [34,40,44,45,46,63]	USA Midwest	1998–2015	2.1 (0–12)	17.7 (3.7–41)
*I. pacificus* infection [48,51,53,54,55,56,57,58,60]	USA Far West	2000–2016	1.2 (0–3.7)	3.9 (0.6–7.1)
*I. scapularis* infection [71,77]	Canada	2011–2020	0.6 (0–0.7)	17.4 (0–33.3)

Human *B. miyamotoi* infection has been noted in most areas where infected ticks have been identified, although more limited in geographic range than tick infection (Figure 2). Explanations for this disparity include the possible absence of a threshold number of *B. miyamotoi* organisms in ticks to effectively transmit infection in some areas; missed diagnosis of human *B. miyamotoi* infections because patients do not seek medical care; or because healthcare workers do not make the diagnosis or do not report *B. miyamotoi* infections to public health authorities [84,85]. *B. miyamotoi* infection is not nationally reportable in the United States and is reportable in only a few states, including Connecticut, Maine, Massachusetts, Minnesota, New Jersey, Vermont, and Wisconsin. The symptoms of *B. miyamotoi* are usually non-specific and make diagnosis difficult. Confirmation of the diagnosis depends on laboratory testing that may not be readily available [7,8]. In contrast, Lyme disease is accompanied in most cases by a diagnostic erythema migrans rash that is usually easy to recognize and Lyme disease is nationally reportable. Nonetheless, the actual number of Lyme disease cases is thought to be about 10-fold greater than the reported number of cases [85]. The discrepancy between diagnosed and undiagnosed infection is probably even greater for *B. miyamotoi*, a tick-borne disease that lacks an easily identifiable clinical marker, such as the erythema migrans rash, and is less well known by health care workers and the general public.

## 2. Clinical Manifestations

### 2.1. General Clinical Course

*B. miyamotoi* symptoms usually begin 14 days (10–18 days) after a tick bite [6]. The most common presentation is a non-specific viral-like illness with fever that may exceed 40 °C, chills, headache, myalgia, fatigue, arthralgia, and gastrointestinal complaints (Table 2). An erythema migrans rash has been noted in about 5 % of cases [5,16,30,86,87,88,89]. The most striking clinical feature of *B. miyamotoi* is relapsing fever with an initial febrile episode followed by a period of wellness and then one or more additional febrile episodes. In the first report of *B. miyamotoi* in humans, about a tenth of 46 Russian *B. miyamotoi* cases experienced two to three episodes of relapsing fever [5]. The average time between relapses was 9 days with a range of 2 days to 2 weeks. In the largest case series of *B. miyamotoi* cases in the US, only 2 of 51 cases (4%) developed relapsing fever [5]. All other case series with 50 or more subjects have been reported from Russia, where about a tenth of patients developed relapsing fever and 5 to 9% had an erythema migrans rash [88,89,90,91]. Differences in the frequency of febrile relapses may be due in part to differences in antibiotic prescribing for a non-specific febrile illness. Frequent empiric use of antibiotics for such patients might clear the infection after the first episode of “relapsing fever” and thus prevent relapses. Such practices will decrease the number of reported cases. Differences in the frequency of relapses also may be due to different strains of *B. miyamotoi* or host immune differences. As many as six febrile relapses over many months have been described for relapsing fever species [2]. 

### 2.2. Coinfection

*Ixodes* ticks transmit six other pathogens besides *B. miyamotoi*, and coinfection in ticks is commonly described. Previous studies have found that coinfection of *B. burgdorferi* with either *Babesia microti* or with *Anaplasma phagocytophilum* are often associated with more severe disease compared with that caused by *B. burgdorferi* infection alone [92,93,94,95]. Human *B. miyamotoi* coinfection with *B. burgdorferi* and/or *B. microti* has been documented [16,30,86,88,89,96]. It is unclear whether *B. miyamotoi* and *B. burgdorferi* coinfection leads to more severe disease in humans, but no significant increase in disease complications has been noted thus far in *B. miyamotoi-B. burgdorferi* coinfected patients.

**Table 2:** Common symptoms of *Borrelia miyamotoi* infection. US case data are derived from 4 studies that enrolled more than 5 cases [30,86,97,98]. An asterix (*) denotes symptoms that were not listed in one of the four studies. Worldwide cases are abstracted from Hoomstra et al. [6].

Symptom	US Cases (85) No. (%) with Symptom	Worldwide Cases (504) No. (%) with Symptom
Fever	80 (94%)	479 (95%)
Chills/rigors	67 (79%)	343 (68%)
Headache	61 (72%)	432 (86%)
Myalgia	60 (71%)	328 (65%)
Fatigue	59 (69%)	197 (39%)
Arthralgia	46 (54%) *	225 (45%)
Abdominal complaints	9 (11%) *	214 (42%)
Relapsing fever	3 (4%)	47 (9%)
Erythema migrans rash	0	21 (4%)

### 2.3. Complications 

Reported complications of *B. miyamotoi* infection include relapsing fever that is reported in an average of 8% (range 4% to 14%) of patients and meningoencephalitis, which is a rare complication [5,30,86,87,88,89,90,91,97,98,99,100,101,102,103,104]. Advanced age and compromised immune status appear to be associated with the development of *B. miyamotoi* meningoencephalitis. Seven cases of *B. miyamotoi* meningoencephalitis (four women and three men) have been reported from Germany, the Netherlands, Sweden, and the United States, with a mean age of 68 years (range 53–80 years) [99,100,101,102,103,105]. Immune suppression was present in five of the cases; three had non-Hodgkin lymphoma, one had rheumatoid arthritis, and one had primary membranous nephropathy. Signs and symptoms included headache, fever, fatigue, confusion, cognitive slowing, memory loss, dizziness, hearing loss, neck stiffness, photophobia, ataxia, facial droop and numbness, and uveitis. The duration from disease onset to hospital admission averaged 11 weeks (range 5 days–9 months). Treatment consisted of ampicillin, ceftriaxone, doxycycline, and/or penicillin, and all but one had initial IV therapy. One patient developed a Jarisch–Herxheimer reaction after IV infusion of ceftriaxone and was switched to IV penicillin. Another experienced persistent facial numbness but the other patients had complete recovery following antibiotic therapy [98]. None of the *B. miyamotoi* meningoencephalitis patients experienced coinfection.

It is possible that one or more additional complications described for other tick-borne relapsing fever *Borrelia* spp. might occur with *B. miyamotoi* infections. These include transfusion-transmitted disease, pregnancy complications and neonatal death, iritis, uveitis, and cranial nerve neuropathy [2]. The potential for *B. miyamotoi* transmission through blood donation has been supported by a few studies. Other relapsing fever species (*Borrelia recurrentis* and *Borrelia duttoni*) were reported to have been transmitted through blood transfusion [106]. *B. miyamotoi* transfusion transmission was studied in a murine model, where mice were transfused with *B. miyamotoi* infected blood [107]. The blood was either transfused fresh (i.e., shortly following collection) or stored for 7 days under blood-banking conditions prior to transfusion. Immunocompetent mice receiving *B. miyamotoi* transfused blood developed transient spirochetemia, while transfused immunocompromised (SCID) mice had motile *B. miyamotoi* spirochetes in their blood for up to 28 days following transfusion. Another study demonstrated similar findings and found that *B. miyamotoi* could remain viable in blood after one month under standard blood banking storage conditions [108]. Studies of blood donors in Austria, California, and the Netherlands have found antibody evidence of previous *B. miyamotoi* infection [109,110,111]. 

Pregnant women infected with relapsing fever *Borrelia*, such as *B. duttoni*, experience severe disease and can transmit the infection to their newborn infants. Spontaneous abortion or perinatal death are common in pregnant women with relapsing fever infection but can be cleared in mother and infant with appropriate antibiotic therapy [2,3,112,113]. One case of *B. miyamotoi* infection with possible Lyme disease coinfection in a pregnant woman has been described. She developed a febrile illness in the 28th week of gestation, was hospitalized, and responded well to IV ceftriaxone therapy. She delivered a healthy child at 37 weeks gestation [113].

## 3. Diagnosis, Treatment, and Prevention

The possibility of *B. miyamotoi* infection should be considered in any patient with a febrile illness who resides in or has recently traveled to a region where Lyme disease is endemic, especially during the late spring, summer, or early fall. Additional clinical findings such as chills, headache, myalgia, and fatigue provide support for the diagnosis but similar symptoms may occur with other *Ixodes*-transmitted diseases and acute viral infections, leading to underdiagnosis and misdiagnosis. Diagnosis requires confirmation using specific laboratory tests that include blood smear, polymerase chain reaction (PCR), and/or antibody determination. *B. miyamotoi* can sometimes be identified by microscopic examination of thin blood smears or a spun sample of cerebrospinal fluid stained with Giemsa or Wright stain. Motile spirochetes may be detected by dark-field or phase contrast microscopy. Several PCR assays have been described for the detection of *B. miyamotoi* DNA in whole blood, plasma, CSF, and tissues, using primers specific for 16S ribosomal RNA and for the *flaB* and *glpQ* genes [7,8]. 

Serologic testing may be useful for diagnosis of *B. miyamotoi*, including a fourfold rise in anti-*B. miyamotoi* IgM and IgG antibody in acute and convalescent-phases of infection. Glycerophosphodiester phosphodiesterase (GlpQ) antigen is produced by *B. miyamotoi* but not by *B. burgdorferi* and has been used in ELISA and Western blot assays to distinguish *B. miyamotoi* from *B. burgdorferi* [7,14,114,115,116]. Although the diagnostic value of the GlpQ antibody assay has been questioned, results of a recent study have confirmed that as a single antigen, GlpQ, remains the primary discriminatory marker for *B. miyamotoi* disease [111,116]. Sensitivity and specificity of *B. miyamotoi* assays can be improved by use of variable membrane protein (Vamp) antigens together with GlpQ antigen [72,114,115,116]. *B. miyamotoi* and other relapsing fever spirochetes share multiple antigens which complicate serodiagnosis of *B. miyamotoi* in relapsing fever endemic areas [29]. *B. miyamotoi* antibody also has been found to cross react against the C6 antigen used in some Lyme disease antibody assays [117]. Patients suspected of having Lyme disease because of typical symptoms and a positive C6 ELISA test may actually have *B. miyamotoi* infection. In such cases, the Lyme disease Western blot assay will be negative. In vitro cultivation of *B. miyamotoi* isolates using specialized media is available at selected research laboratories. 

Although no prospective trials have been carried out to evaluate antibiotic treatment for *B. miyamotoi* disease, case series and case reports have indicated that treatment of *B. miyamotoi* infection with the same antibiotics that are used for Lyme disease are effective in clearing symptoms and infection [6,7]. These include doxycycline, tetracycline, erythromycin, penicillin, and ceftriaxone. Physicians should be aware of the small possibility of Jarisch–Herxheimer reaction following the first dose of antibiotic, primarily for those receiving intravenous antibiotic. 

*B. miyamotoi* infection can best be prevented by avoiding areas where *B. miyamotoi*-infected ticks are found [118,119]. These include wooded, brush, and tall grass areas in *B. miyamotoi*-endemic regions. Landscape alterations that limit tick habitat include maintaining a well cut lawn, eliminating leaf litter, spraying yards with tick-repellant (either natural or synthetic products), and separating lawn from wooded areas using a wood chip or pebble barrier [118,120]. Personal protective measures include wearing long pants and long sleeved shirts with pants tucked into socks, and use of acaracides on self (DEET) or clothing (Permethrin). Tick checks and rapid removal of embedded ticks using tweezers and saving them for subsequent species identification are useful [121,122]. Tick-bite antibiotic prophylaxis is available for Lyme disease, but the effect of antibiotic prophylaxis to prevent *B. miyamotoi* infection is unknown. No *B. miyamotoi* vaccine is available.

## 4. Conclusions

*Borrelia miyamotoi* disease is an emerging tick-borne infection caused by a relapsing fever spirochete and transmitted by the same hard-bodied ticks that transmit Lyme disease, babesiosis, and human granulocytic anaplasmosis. It is found worldwide and causes a febrile illness that can sometimes relapse and rarely causes meningoencephalitis, but the full extent of the health burden of *B. miyamotoi* has yet to be determined.

## Data Availability

Not applicable.
